# Laparoscopic and Robot-Assisted Hepatic Surgery: An Historical Review

**DOI:** 10.3390/jcm11123254

**Published:** 2022-06-07

**Authors:** Atsushi Shimizu, Miwa Ito, Alan Kawarai Lefor

**Affiliations:** Department of Surgery, Jichi Medical University, Shimotsuke 329-0498, Tochigi, Japan; ashimizu@jichi.ac.jp (A.S.); m16007mi@jichi.ac.jp (M.I.)

**Keywords:** liver, laparoscopy, robot-assisted surgery

## Abstract

Hepatic surgery is a rapidly expanding component of abdominal surgery and is performed for a wide range of indications. The introduction of laparoscopic cholecystectomy in 1987 was a major change in abdominal surgery. Laparoscopic surgery was widely and rapidly adopted throughout the world for cholecystectomy initially and then applied to a variety of other procedures. Laparoscopic surgery became regularly applied to hepatic surgery, including segmental and major resections as well as organ donation. Many operations progressed from open surgery to laparoscopy to robot-assisted surgery, including colon resection, pancreatectomy, splenectomy thyroidectomy, adrenalectomy, prostatectomy, gastrectomy, and others. It is difficult to prove a data-based benefit using robot-assisted surgery, although laparoscopic and robot-assisted surgery of the liver are not inferior regarding major outcomes. When laparoscopic surgery initially became popular, many had concerns about its use to treat malignancies. Robot-assisted surgery is being used to treat a variety of benign and malignant conditions, and studies have shown no deterioration in outcomes. Robot-assisted surgery for the treatment of malignancies has become accepted and is now being used at more centers. The outcomes after robot-assisted surgery depend on its use at specialized centers, the surgeon’s personal experience backed up by extensive training and maintenance of international registries. Robot-assisted hepatic surgery has been shown to be associated with slightly less intraoperative blood loss and shorter hospital lengths of stay compared to open surgery. Oncologic outcomes have been maintained, and some studies show higher rates of R0 resections. Patients who need surgery for liver lesions should identify a surgeon they trust and should not be concerned with the specific operative approach used. The growth of robot-assisted surgery of the liver has occurred in a stepwise approach which is very different from the frenzy that was seen with the introduction of laparoscopic cholecystectomy. This approach allowed the identification of areas for improvement, many of which are at the nexus of engineering and medicine. Further improvements in robot-assisted surgery depend on the combined efforts of engineers and surgeons.

## 1. Background

This review will highlight the development of robot-assisted liver surgery by progressing through the development of liver surgery, laparoscopic surgery, laparoscopic liver surgery, robot-assisted surgery (including robot development, training and simulation, and non-hepatic robot-assisted surgery), and finally, to robot-assisted liver surgery. This will be accomplished with a historical perspective in each phase of the development in a manner similar to a previous report of robot-assisted surgery of the pancreas [1]. Open surgery has been performed for thousands of years on various parts of the body. The surgical approach to the human body changed dramatically in 1987 with the introduction of modern laparoscopic general surgery (also referred to as minimally invasive surgery).

Many terms have been used to describe surgical applications of robots, such as robot-assisted minimally invasive surgery, robotic surgery, robot-assisted laparoscopic surgery, and others. This review will discuss the various approaches under the single term robot-assisted surgery. Nearly all robot-assisted surgery performed today uses a “master”–“slave” system. The surgeon is performing the surgical procedure with assistance from the robot [2], and therefore this type of procedure is called robot-assisted surgery. Robotic surgery refers to autonomous surgery performed by a robot which is of great interest and importance but is not yet in clinical use. Robot-assisted surgery can be viewed as the peak of a three-story building, with laparoscopic surgery in the middle, with everything based on a foundation of routine open surgery. History will be the central guiding force of this review as it moves upward in this three-story building, starting with open hepatic surgery, then to non-hepatic laparoscopic surgery and laparoscopic hepatic surgery, and finally to general robot-assisted surgery, and then to robot-assisted surgery of the liver. A discussion of a range of procedures is included to facilitate gaining a historical perspective.

## 2. Liver Surgery

**Figure 1:**
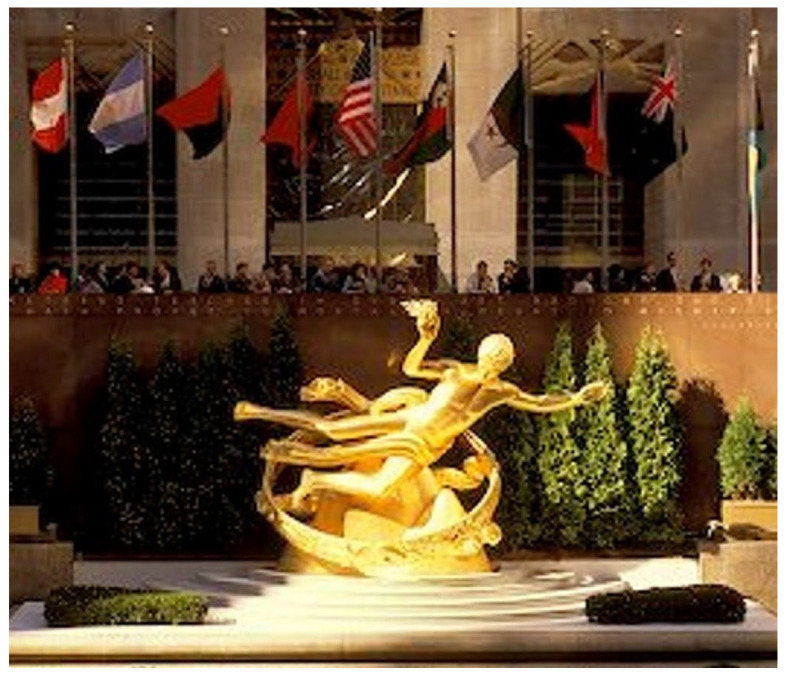
The statue of Prometheus at Rockefeller Center in New York City (Photograph by the author).

The liver has been an organ of fascination for thousands of years. It is of great interest that the ancient Greeks knew about liver regeneration, as described in the story of Prometheus, whose liver was devoured daily by an eagle (Figure 1).

When the eagle returned, the liver had regrown to full size. The regenerative ability of the liver, combined with the fact that this large organ with hundreds of known physiologic functions has no known anatomical areas of specialization, is truly a wonder. These two facts demonstrate the unique nature of the liver in the human body. In the early 1500s, Galen convinced the scientific community that the liver was the principal organ of the body, although this was later challenged based on the teachings of Vesalius.

Moving ahead to modern times, the first liver resections were performed on patients with traumatic injuries resulting from combat in the seventeenth and eighteenth centuries. The first planned non-traumatic liver operation was performed in 1888 by Langenbuch [3]. The Australian surgeon Pringle introduced the maneuver for in-flow occlusion that carries his name in 1908. The origins of modern liver surgery started in the 1950s with isolated publications, but there was no defined technique at that time for liver resection. A milestone in liver surgery was reached in 1956 when Couinaud published an anatomical guide to the liver, including the segmental anatomy, which bears his name. Despite these anatomic descriptions, surgical practice was limited, and only major liver resections were usually performed. The practice of liver surgery was altered in the 1980s with the development of diagnostic ultrasound and the concept of navigating the liver on the basis of anatomy in a 1982 publication [3]. The introduction of intraoperative ultrasound in 1984 by Makuuchi has also advanced the techniques used in hepatic surgery.

Another major force leading to advances in liver surgery was the growth of liver transplantation, which was started in 1963 by Starzl. Advances in this field have been rapid and driven by many other changes, such as the development of anti-rejection medications and other approaches. The control of intraoperative bleeding in liver surgery remains a challenge but one which has been met with a wide variety of techniques that are well-known to the experienced liver surgeon. All of these developments throughout history have led to a drastic reduction of operative mortality from early reports as high as 20% to current rates of less than 1% mortality for many hepatic procedures [3].

## 3. Laparoscopic Surgery

Moving toward robot-assisted hepatic surgery, the next major historical milestone is the rapid popularization of laparoscopic surgery (as used here, this term always refers to non-robot-assisted minimally invasive surgery), one form of minimally invasive surgery. While laparoscopic surgery became widely adopted by general surgeons in 1989, laparoscopic surgery did have a previous history but was limited mostly to gynecologic procedures. In the late 1980s, the right upper quadrant minilaparotomy for cholecystectomy was gaining in popularity. The first laparoscopic cholecystectomy was performed by Mouret in France in 1987 [4]. This was then performed in the United States (by Dr. Eddie Joe Reddick). Laparoscopic surgery was actively prohibited at some major university centers in the United States. Most of the initial procedures were performed at non-university medical centers and, after some time, became accepted at university centers. The first laparoscopic cholecystectomy performed at a university medical center in the United States was at the University of Maryland (also the origin of the “Maryland Dissector”) in November 1989 by Karl Zucker, Robert Bailey, and John “Jack” Flowers.

Early critics suggested that laparoscopic cholecystectomy should only be done at specialized centers [5]. Laparoscopic surgery forever changed General Surgery and became a widely used surgical approach. This revolution did not start in research laboratories as many changes in medicine do. There was very little data, but despite this, the procedure rapidly spread [5]. The financial benefits in healthcare powered this rapid growth. Patients demanded laparoscopic procedures after watching programs on the evening television news. The instruments used to perform laparoscopic surgery were new in 1987, in particular the video-laparoscope and camera/display, which are important because they allow the surgical team to share one view of the operative field. Many training courses were offered around the world. With the rapid ascent and widespread acceptance of laparoscopic cholecystectomy, interest in minilaparotomy for cholecystectomy waned just as quickly. As the use of laparoscopic cholecystectomy increased, reports of bile duct injuries raised significant concerns in the surgical and medico-legal communities, in part because they were not so common before the introduction of laparoscopic cholecystectomy. These were felt to be a result of the “learning curve” and are less often mentioned today as a specific consequence of minimally invasive surgery. Despite this, iatrogenic bile duct injuries remain an important morbidity that must be considered [6].

After the introduction of laparoscopic surgery, it was not long before most abdominal operations were performed using these techniques. Instruments and methods used in abdominal procedures were adapted to minimally invasive surgery in the chest. For example, thoracoscopic lung resection is now the standard. Minimally invasive surgery is routine for procedures such as colon resection, Nissen fundoplication, appendectomy, and splenectomy.

Minimally invasive surgery was later used in the treatment of patients with a variety of malignancies, which was avoided early in its history. Once malignancies were resected, there were several reports of lesions, such as port-site recurrences, which had been unknown prior to this [7]. These reports raised issues about oncologic safety and long-term outcomes. The majority of these questions have been resolved adequately. The minimally invasive surgery revolution is certainly remarkable. The widespread use of minimally invasive surgery has resulted in improved patient outcomes and changes throughout the healthcare industry and has also fueled the growth of other industries.

## 4. Laparoscopic Hepatic Surgery

With the rapid advances and growth in laparoscopic surgery starting in 1989, it is not surprising that the liver soon became an organ of interest for laparoscopic surgeons. Initial reports of laparoscopic hepatic resections for benign tumors were made in 1991 [8]. Laparoscopic liver biopsy was reported in 1993, performed with a stapling device [9]. Laparoscopic hepatic resection was initially reported in 1995 by Rau, who described the resection of six tumors with the water jet dissector, and also by Cuesta, who described the resection of two benign tumors, and by Hashizume, who described the resection of a hepatocellular carcinoma [10]. Laparoscopic left lateral sectionectomy was first reported in 1996, and formal hemi-hepatectomies in 1998. There have been many studies of laparoscopic hepatic resection, and consequently, there are also a large number of meta-analyses comparing laparoscopic hepatic resection with open surgery. The history of this rapidly advancing field has been reviewed extensively [8,11]. In this section, we endeavor to briefly introduce the techniques of laparoscopic hepatic resection without attempting to be encyclopedic in the coverage.

The rapid advances in laparoscopic hepatic surgery are the result of innovative advances in surgical approaches with the development of new techniques [12]. Early laparoscopic surgery of the liver progressed using many of the approaches that had been developed for open liver resection, such as clamp-crush techniques, portal dissection, and inflow occlusion [12]. The approach to the liver has evolved with the widespread use of stapling devices and coagulation devices which has reduced the need for hilar dissections. It is expected that advances in laparoscopic hepatic surgery will continue. While early laparoscopic approaches to the liver were limited to wedge resections and small surface lesions, this has rapidly advanced to performing a wide range of resections, including left lateral segmentectomy, right lobectomies, and living-donation for liver transplantation. The importance of basic technical considerations for this procedure, such as surgeon positioning, port placement, liver mobilization, intraoperative tumor assessment, vascular control, and the use of sealants, have been described [13].

Several important caveats have been pointed out. The indications for laparoscopic liver resection are the same as for open surgery. The only limiting factor is technical feasibility. Bleeding can be a major intraoperative concern. Hand assistance is useful in selected cases [14]. In part due to the rapid advances in this field and its rapid adoption worldwide, there have been International Consensus Conferences conducted by leaders in the field to provide guidelines for conducting these operations. The Louisville statement from the first consensus conference was issued in 2009 [15], and a preparatory paper for the Second Conference was issued in 2014 [11]. The second consensus conference was conducted in Morioka, Japan, and a report was published in 2015 [16]. Seventeen questions were addressed at this conference. Seven of these questions were focused on outcomes that evaluate the risks and benefits of laparoscopic hepatic resection. The answers to these questions were determined using a consensus conference model. The literature and expert opinions were weighed by a nine-person panel, which evaluated outcomes using multiple comparators. The panel also graded laparoscopic hepatic resection by the Balliol Classification. They concluded that minor laparoscopic hepatic resections had become standard practice and that major hepatic resections were still being investigated. The cautious adoption of major laparoscopic hepatic resections was recommended. The final 10 questions identified by the panel were directed at technical aspects of the procedure and recommendations based on existing literature reviews and expert opinions. The questions led to recommendations for preoperative evaluation, hemostasis, techniques for transection, anatomic approaches, and equipment. The panel recognized the need for a formal education structure to train surgeons interested in performing major laparoscopic hepatic resections because of the “steep” learning curve.

The consensus conference concluded that patient protection is most important [17]. The conference recommended several areas as critical for patient protection: (I) a prospective registry; (II) a difficulty scoring system to select appropriate patients; (III) the creation of a formal education structure. An online prospective registry system for the calculation of difficulty was created in Japan after the conference to advance further safe development of laparoscopic hepatic resection.

In addition to guidelines from these important international consensus conferences [18], experience-based guidelines for laparoscopic hepatic resection have also been described [19]. Evidence supporting laparoscopic hepatic resection comes from a number of types of studies, including comparative studies, meta-analyses, and case series. While there is little strong evidence, data in the existing literature confirms the safety, feasibility, and benefits of laparoscopic hepatic resection compared to open procedures [19]. Indications for laparoscopic hepatic resection are the same as those for open surgery and include both benign and malignant lesions (primary/metastatic) and living donor liver harvesting [19]. Currently, resection of lesions located in anterolateral segments and left lateral sectionectomies are performed laparoscopically on a routine basis in specialized centers. Resection of lesions located in the posterosuperior segments and major resections are feasible but remain technically demanding and should be performed by experienced surgeons at specialized centers [19]. Laparoscopic hepatic resection is considered safe for the resection of malignant lesions and may offer short-term benefits over open resection. Oncologic results, such as the status of resection margins and long-term survival, were similar to results with open resection. One of the important features of recent guidelines in laparoscopic hepatic resection, especially in contrast to early laparoscopic surgery, is the inclusion of a focus on education and training in these new techniques.

To examine the pervasiveness of this technique, a 2016 report described the results of a survey of liver resections performed between 2008 and 2013 [20]. Data was collected from 27 respondents, all of whom participated in the second international consensus conference. From 2008 through 2013, 11,712 liver resections were performed at 27 centers. Of these liver resections, the laparoscopic approach was used in 32% (n = 3765), while open resection was used for 68% (n = 7947). The ratio of laparoscopic resections taken in all left lateral sectionectomy procedures (62%) is higher compared with that of all laparoscopic hepatic resections (32%), all minor liver resections (36%), and all major liver resections (25%).

In an early report from a single institution, authors reviewed 21 resections in 17 patients, including 5 for malignant lesions and 12 for benign lesions [21]. There was a mean of 1.4 lesions resected at an average size of 7.6 cm in an average operating time of 2.8 h. The mean blood loss was 288 cc. Investigators reported complications including biliary leak (n = 1), re-operation for hemorrhage (n = 2), and death due to hepatic failure (n = 1). The mean length of stay was 2.9 days. When compared to 100 patients from the same institution who underwent open resection of benign liver tumors, there was significantly greater mean blood loss (485 cc), mean operative time (4.5 h), and mean length of stay (6.5 days). It is instructive to examine this data from a very early series [21].

In an early systematic review of laparoscopic hepatic resections, authors reviewed 28 studies, including 703 patients [22]. The most common procedures were wedge resection (35%), segmentectomy (22%), and left lateral sectionectomy (21%). Right hepatectomies represented less than 4% of the resections. The conversion rate was 8.1%, and the complication rate was 17.6%. The overall mortality rate was 0.8%, and the median (range) hospital length of stay was 7.8 days. Eight case–control studies were included in this review. While some had significant reductions in hospital length of stay, there was no reduction in complication or mortality rates associated with laparoscopic hepatic resections [22].

As of 2009, there had been no randomized trials with all of the data reported as case series [14]. There were only 12 studies until that time that reported 50 or more cases, and these studies had a wide range of percentage of cases for the treatment of malignancies. Until that time, feasibility was the major criterion studied. These authors suggest that laparoscopic resection is not indicated for lesions larger than 5 cm. The conversion rate reported was 5–15%. In this meta-analysis, there were 10 deaths reported from over 2000 laparoscopic hepatic resections. A total of 12 case-control studies were reported comparing laparoscopic and open hepatic resections. They report that laparoscopic liver resections were associated with reduced blood loss and similar operating times [14].

A 10-year experience with totally laparoscopic hepatic resections was published from a single institution [23]. These authors describe 82 patients who underwent laparoscopic hepatic resection for liver metastases (n = 39), primary hepatocellular carcinoma (n = 37), and benign lesions (n = 6). Procedures performed include 69 wedge resections, 11 left lateral segmentectomies, and 2 thoracoscopic wedge resections. Nine patients underwent simultaneous laparoscopic resection of liver metastases and the primary colorectal tumor. The median operating time was 177 (range 70–430) min, and the mean estimated blood loss was 64 mL (range 1–917). The median tumor size was 25 mm, and the median margin of resection was 6 mm. The median length of stay was 9 days. The 5-year survival rate after surgery for hepatocellular carcinoma was 53% and for colorectal metastases was 64%. The authors conclude that laparoscopic hepatic resection is safe for resection of a variety of primary and secondary hepatic tumors [23].

In 2012, a meta-analysis of 15 studies included 1002 patients, 446 laparoscopic and 556 open hepatic resections [24]. This study found no significant differences between the two groups for survival and no difference in results for hepatocellular carcinoma or colorectal metastases. There was a slightly improved survival three years after resection for patients who underwent laparoscopic resection of colorectal metastases.

A series of 166 laparoscopic hepatic resections performed between 1996 and 2007 were reported, including 100 (60%) for malignant tumors (64 HCC, 3 cholangiocarcinoma, 33 metastases) and 66 for benign lesions [10]. The number of resections performed for benign lesions remained stable while those for malignant lesions increased over time. There were 31 major hepatic resections, 56 left lateral sectionectomies, 28 segmentectomies, and 51 tumor resections. The mortality was 0%, and the morbidity was 15%. The median blood loss was 200 mL, transfusions were given to 9 patients, and the median operating time was 180 min. The authors emphasized the importance of selecting appropriate patients and surgical techniques developed with experience [10].

One of the largest and earliest series of laparoscopic hepatic resections was reported in 2008, and the authors reviewed 590 minimally invasive hepatic procedures performed during 489 operative procedures [25]. These resections included hepatocellular carcinomas (n = 210), colorectal carcinoma (n = 40), other malignancies (n = 62), and benign lesions (n = 176). The conversion rate was 2% overall. Patients were divided into three groups, including laparoscopy/biopsy, laparoscopic radiofrequency ablation, and laparoscopic resection. Complication rates for radiofrequency ablation and resection were comparable, but complications and mortality were higher for cirrhotic patients compared with non-cirrhotic patients. The authors conclude that minimally invasive hepatic surgery is a reasonable alternative to open hepatic surgery. They also found superiority of laparoscopic hepatic resection over radiofrequency ablation for interim survival rates [25].

A large number of studies have been written, including mostly case-control studies, comparing laparoscopic liver and open liver resections. Many of these studies are worthwhile to review. One of the studies which took a different approach to this comparison was published in 2020 as a comparison of economic and clinical outcomes [26]. Patients undergoing liver resection from 2015 to 2018 and entered into a database were analyzed with propensity score matching to evaluate complications, in-hospital mortality, readmission rate, discharge to an extended care facility, operating time, length of stay, and total cost. Of eligible open liver resection (n = 3349) and laparoscopic liver resection (n = 1367) patients, 1261 were propensity score-matched at a 1:1 ratio. After matching, laparoscopic liver resection was associated with lower rates of complications (intestinal obstruction: 3.6% vs. 6.0%; respiratory failure: 5.5% vs. 10.9%; bleeding: 8.2% vs. 17.4%; and pleural effusion: 1.9% vs. 4.9%), in-hospital mortality (0.5% vs. 3.0%), 90-day readmission (10.4% vs. 14.3%), discharge to extended care facility (6.9% vs. 12.3%), operating time (257 vs. 308 min) hospital length of stay (4.3 vs. 7.2 days), and hospital costs (USD 19,463 vs. USD 29,119) (all *p* < 0.001). The authors conclude that laparoscopic hepatic resection is associated with a lower risk of complications and reduced resource utilization compared with open liver resection [26].

Until this point, we have discussed several approaches to laparoscopic hepatic resection, which may be considered routine. In addition, other approaches have been developed to handle specialized situations. In a study comparing laparoscopic, hand-assisted laparoscopic, and laparoscopy-assisted methods, a literature review was conducted, and a total of 29 articles were analyzed [27]. It was concluded that laparoscopic major hepatic resections achieve similar patient and economic outcomes compared with open liver resections in selected (noncirrhotic) patients. Surgeon experience affects the results and a learning period by the surgeon is essential. Laparoscopic repeat liver resections for hepatocellular carcinoma have been evaluated [28]. This large review accrued data from 42 centers around the world, and the authors concluded that repeat laparoscopic liver resection is a reasonable option in selected patients. Laparoscopic hepatic resection has been described in several ways, including pure laparoscopic, hand-assisted laparoscopy, and a hybrid approach that starts as pure laparoscopic or hand-assisted, and a resection is then performed through a mini-laparotomy. These three were considered in a literature review along with open resection [29]. The authors included nine studies pertaining to hand-assisted live resection and nine related to the hybrid technique. The authors concluded that most centers use a combination of pure laparoscopic approaches, hand-assisted, and the hybrid approach. The authors conclude that there is insufficient evidence to determine if any of these four approaches (pure laparoscopic, hand-assisted, hybrid technique, and open laparotomy) is superior. Hand-assisted laparoscopic surgery and the hybrid technique may help to overcome some of the problems associated with laparoscopic hepatic surgery and may be less invasive than surgery.

Laparoscopic hepatic resection has become a widely used technique that has been developed carefully with input from many institutions around the world, and this development has been guided by evidence-based approaches. In light of this rapid and widespread development, leaders in the field formed the International Laparoscopic Liver Society in 2016 [30]. This is an important organization to which we can look for further developments in this rapidly changing field (https://www.lap-liver.com/). The goals of the organization are to organize biennial congresses, coordinate international registries, provide a collaborative forum for surgeons, and educate surgeons on these techniques. This is a valuable resource for surgeons in the field or wishing to start in this exciting and expanding field.

## 5. Robot-Assisted Surgery

The word robot was first used by the playwright Capek in 1921 for the play *Rossom’s Universal Robots*. Robots have long been objects of fascination and used widely in literature. “Robot” is from a Czech word meaning “forced labor”. Robots are widely used in many fields, especially in manufacturing, which simplifies production as well as facilitates the exploration of hazardous areas. The widespread application of robots in medicine could not have happened without recent developments in microprocessor technology.

Surgical robotics started to be considered broadly after the adoption of laparoscopic cholecystectomy. Until then, there were only highly specialized robots. Robot-assisted surgery is a direct outgrowth of conventional minimally invasive surgery. The AESOP device was invented by Computer Motion Inc. (Santa Barbara, CA, USA) in 1983 to automatically position endoscopic devices [31]. The first iteration of the da Vinci was developed by Intuitive Surgical Inc. (Sunnyvale, CA, USA) in 1997. It rapidly became used on a widespread basis in the United States in about the year 2000. The Zeus robot was introduced by Computer Motion in 2001. Following this, the Computer Motion and Intuitive Surgical companies merged. The da Vinci robot is surely the most commonly used surgical robot used in the world today. Robots were of considerable interest to the military to allow telerobotic surgery near the battlefield.

The da Vinci surgical robot is sometimes referred to as a “master–slave” system. The surgeon sits at a console in the operating room [32]. The instruments are attached to the “slave” unit and placed into the patient in a manner similar to that used for laparoscopic surgery and then attached to the robot. The third component of the da Vinci robot is the vision system. The surgeon manipulates instruments by moving hand-operated controls at the console. For this reason, it is referred to as robot-assisted surgery. The motion of each instrument is controlled by the surgeon but indirectly by electromechanical coupling. The instruments move and follow the surgeon’s hands which directly move the electronic joysticks. The instruments move relative to the camera as the surgeon’s hands move relative to the eye, which enhances hand-eye coordination in robot-assisted surgery. The computer control of the da Vinci system includes tremor filtering, motion scaling, and an articulated wrist. The da Vinci system initially costs approximately USD 3M, with about USD 200,000 in maintenance costs annually.

Using robots in surgery is an extension of conventional minimally invasive surgery. Most of the operations that were reported to have been performed using robot-assisted technology had already been reported to have been performed laparoscopically. This represents a progression from open surgery to laparoscopic surgery and then to robot-assisted surgery. The development of robot-assisted surgery has been motivated by attempts to overcome the limitations associated with laparoscopic surgery as well as to improve outcomes [33]. Early in their development, the advantages of the use of surgical robots were clear [31]. In contrast to typical laparoscopic surgery, robot-assisted surgery enables three-dimensional imaging and gives the surgeon improved dexterity with up to seven degrees of freedom in the instruments. Robots also smooth the motion of the instruments, which eliminates the effect of hand tremors and allow motion scaling for instrument motion. Surgical robots facilitate conducting telepresence surgery which has been performed across the Atlantic Ocean [31]. The surgeon sits comfortably at the console, which reduces fatigue. Surgical robots facilitate conducting repetitive motions and working in deep body cavities, such as suturing in the pelvis.

In routine open surgery, surgical instruments are moved directly by the hand of the surgeon, and the same is true in laparoscopic surgery. Robot-assisted surgery takes a totally different approach where the surgeon’s hand manipulates an electronic controller. The signals from the controller are transmitted electronically to actuators which move the instruments. The indirect motion of instruments facilitated by the robot allows the computer to filter out tremors and increase the range of motion limited by the movement of the human hand. This also may represent a watershed moment in surgical education with the ability to collect terabytes of intraoperative hand motion data from the robot for analysis and characterization of surgical skills.

Surgeons and the general public are fascinated by robot-assisted surgery. Early in the promulgation of laparoscopic surgery, surgeons who were not performing laparoscopic surgery had fewer patient referrals. This same phenomenon is happening to some degree with robot-assisted surgery. When treating patients for non-malignant conditions, there are increased concerns about the added costs incurred by the use of the robot and longer operating room setup time.

Robots used in medical practice are different from robots used for industrial purposes. Diaz and colleagues analyzed the need for robots in medicine to guide future development [34]. They describe a number of clinical needs such as intervention time, setup time, footprint in the operating room, cost reduction, improved decision making, and data integration. These authors highlight technical requirements such as shape, weight, and size, which all need to be reduced. They also highlight the need for more degrees of freedom in the instruments, improved motion resolution, retraction of tissue, haptic feedback, improved spatial orientation, wireless functionality, fewer instrument exchanges, and instrument flexibility. This review serves as a clear explanation of the interface engineering-robot interface and identifies areas for future development.

## 6. Training, Assessment, and Simulation in Robot-Assisted Surgery

Robot-assisted surgery is still in its infancy. Perhaps as a result of the seemingly uncontrolled activity during the initial adoption of laparoscopic surgery with little structure in training programs, leaders in robot-assisted surgery are designing carefully structured educational programs. Some of these efforts are led by international working groups which are designing programs for training in all aspects of minimally invasive surgery [35]. International conferences are being held to specifically focus on educational issues. A learning curve has been clearly demonstrated for minimally invasive surgery of the liver, and there is concern that low case volumes at some institutions may adversely affect outcomes. The consensus of the group was that a change from the old paradigm of “see one, do one, teach one” is essential. Competency after training should be based on mastering a well-defined skillset, and this training should be based on simulation and tissue-based training. The development of specialized centers to train practitioners using a standardized approach and proctoring is important. Reporting of outcomes is expected as part of training.

Programs for training in robot-assisted surgery have become widespread in the United States. A survey of general surgery residency program directors evaluated opinions about training for robot-assisted surgery [36]. Program directors from a variety of training programs were surveyed. Formal training for residents in robot-assisted surgery was provided by 74% of programs, and 63% also employed simulation training. Most program directors agreed that more time should be spent on training in robot-assisted surgery, and 63% wanted the inclusion of formal training programs as part of the general surgery curriculum. This exposure would ideally start as early as the first year of residency training.

The widespread use of robot-assisted minimally invasive surgery brought with it hope for improved clinical outcomes. Until the present time, there has been little proven clinical benefit from using robot-assisted minimally invasive surgery, especially compared to laparoscopic surgery, despite the increased cost and operative time associated with robot-assisted minimally invasive surgery [37]. It is hoped that further refinement of the technology and its use will benefit patients directly. The widespread use of robot-assisted minimally invasive surgery, however, demands changes in education. The ability to collect detailed hand motion information is a major change due to robot-assisted surgery and may be a significant contribution to surgical education. While it seems reasonable to assess basic manipulation skills based on hand motion, the availability of such data has been extremely limited. The use of hand motion data to assess basic manipulation skills began with the use of external sensors, but these are difficult to use in clinical practice [38]. The da Vinci Surgical System for robot-assisted minimally invasive surgery (Intuitive Corp., Sunnyvale, CA, USA) collects kinematic data (instrument motion) at frequencies up to 100 Hz, including 192 data values at each time point [39].

Studies of hand motion during robot-assisted minimally invasive surgery are needed but difficult to conduct. Kinematic data from the da Vinci surgery system has been used for skill assessment in clinical surgery. Recent studies have examined correlations between automated performance metrics and kinematic surgical skill [40,41]. These investigators have found that expert surgeons use their dominant hand more than novices do [40]. The same group has found that even after achieving expertise, surgeons continue to improve [41]. There are ongoing studies to examine the correlation between kinematic parameters and clinical outcomes.

Simulation, especially to teach laparoscopic surgery, developed with a wide range of equipment ranging from cardboard boxes to computer-based virtual reality simulators. There have been studies comparing video games to surgery simulators [42,43]. One of the key features that makes video games successful is accessibility, which may also be important in the design of surgery simulators [44]. Virtual reality for surgical simulation has been made possible by advances in computer technology, including processor speed and inexpensive memory. Virtual reality training has been used for training in laparoscopic surgery. A large meta-analysis reviewed the role of virtual reality training compared to other modalities and found that it is most beneficial for trainees with no or little experience [45]. The authors could not conclude that virtual reality results in improved skill transfer from training to the operating room. In another meta-analysis of 8 trials and 109 trainees, investigators found that virtual reality training shortened operating time during surgery after the training [46]. In three trials comparing virtual reality simulation training to box or video trainers in a review, training was considered equal with either type of simulator [47]. Simulators can be designed to provide accurate hand motion data and may be an efficient way to obtain such data.

## 7. Non-Hepatic Robot-Assisted Surgery

Readers interested in a detailed view of the current status of robot-assisted surgery are advised to review an evidence-based report current of 2012 [48]. When laparoscopic surgery became well accepted in general surgery, laparoscopic cholecystectomy was the only procedure performed by many surgeons. Since robot-assisted surgery is somewhat related to laparoscopic surgery, robot-assisted surgery has quickly evolved to be applied to many different kinds of operations. There is no one operation or organ that is particularly favored by surgeons performing robot-assisted surgery. All of the operations being done using a robot-assisted approach had been done laparoscopically beforehand.

The role of robot-assisted surgery in the management of patients with malignancies has been discussed [2]. The authors suggest that robot-assisted surgery may allow the performance of more complex and difficult procedures because of the improved vision and dexterity that robot-assisted surgery offers. This may lead to more accurate resection margins and improved lymph node resections. It is currently unknown if this will translate to improved clinical outcomes.

Although this discussion is mainly to describe the robot-assisted treatment of hepatic lesions, there has been a great deal of experience using robot-assisted surgery for bariatric procedures, which is worthy of review. It is hoped that improved vision and dexterity associated with robot-assisted surgery will improve the outcomes compared to laparoscopic bariatric surgery. Bariatric procedures are becoming more commonly performed due to the incidence of obesity. Robot-assisted Roux-en-Y gastric bypass was reported first in 1999 [49]. Early studies showed good outcomes and showed that most surgeons have a learning curve of about 10–15 procedures. This is less than that shown for the laparoscopic version of the operation. When performing laparoscopic gastric bypass, the anastomoses had been performed with a stapler, but many surgeons perform a sutured anastomosis when performing robot-assisted surgery due to enhanced ability to suture [32]. A meta-analysis of laparoscopic versus robot-assisted bariatric surgery was reported [49] and reviewed 14 comparative studies with heterogeneity among operative details. The change from stapled to sewn anastomoses was followed by a decreased leak rate in the robot-assisted patients in some of the papers. Conversion rates are lower for robot-assisted surgery, but this may be due to the learning curve. Some studies reported a lower rate of postoperative strictures after robot-assisted surgery. Most studies in this meta-analysis described longer operating times with robot-assisted surgery.

Robot-assisted surgery has been used extensively in fields other than General Surgery. Studies have described robot-assisted surgery of the head and neck, including the pharynx, larynx, nasopharynx, sinuses, and anterior skull base [50]. Radical neck dissections have been performed using robot-assisted techniques. This review describes a number of clinical trials for head and neck robot-assisted surgery. These authors discuss costs as well and state that the costs of robot-assisted laryngeal surgery performed are 90% higher than conventional procedures. This large difference is due to the increased cost of instrumentation.

There are a number of studies of robot-assisted surgery of the thyroid. A study of the learning curve for robot-assisted thyroid surgery was reported [51] as a prospective multi-center study with four experienced endocrine surgeons. A total of 644 thyroid resections were reviewed. Results were compared based on the surgeon’s experience, and they determined that the learning curve for total thyroidectomy is 50 cases, and for subtotal thyroidectomy, it is 40 cases.

Robot-assisted prostatectomy for cancer has become popular among both surgeons and patients. Some patients demand this approach when they need a prostatectomy. However, available data for robot-assisted prostatectomy for cancer does not uniformly show a benefit to patients. There are very few randomized prospective trials for this procedure. In one randomized trial designed to evaluate short-term outcomes, investigators found similar outcomes for both open and robot-assisted radical prostatectomy [52]. The robot-assisted surgery group had some benefits, such as earlier hospital discharge, reduced blood loss, fewer adverse events, and improved postoperative quality of life. These investigators continued to follow the patients for long-term oncologic outcomes [53]. Finally, the study concluded that robot-assisted surgery has outcomes equivalent to open surgery at 24 months. The authors state that the observed benefits of robot-assisted surgery are related to the fact that it is a minimally invasive procedure. In a subsequent commentary, it was concluded that patients should choose a surgeon they trust rather than deciding based on the particular surgical approach [54].

Robot-assisted distal gastrectomy for gastric cancer has been widely reported. A study compared 109 patients after robot-assisted distal gastrectomy with 160 patients after laparoscopic distal gastrectomy [55]. All lesions were stage cT1, and the two groups were otherwise similar for most characteristics. The authors reported a tendency (*p* = 0.112) toward reduced infectious complications in patients after robot-assisted surgery. Injuries to the tail of the pancreas are well known after gastric surgery, which can result in an amylase leak. There were significantly decreased amylase levels in drains of patients after robot-assisted surgery. The results of this report show that robot-assisted distal gastrectomy is comparable to laparoscopic resection. Randomized prospective trials are needed in this area.

Robot-assisted colon resections for cancer have been reported extensively. A single port approach to further reduce postoperative port site pain with a superior cosmetic result has also been reported. A meta-analysis of single port resections of colon cancer was reported [56]. Current studies show that this approach is safe and feasible, but the quality of evidence in studies performed so far is low. New developments in robot-assisted surgical technology are needed to facilitate single-port surgery.

This brief overview of non-hepatic robot-assisted surgery shows that a wide range of surgical procedures is being performed with robot-assisted techniques. Despite the fact that there were many early concerns about laparoscopic resection of malignancies, especially with reports of unusual developments such as port-site metastases, these concerns have been alleviated with greater experience. Similarly, robot-assisted surgery is used freely for the treatment of patients with malignancies.

## 8. Robot-Assisted Hepatic Surgery

Robot-assisted hepatic surgery has rapidly become a widely used modality for liver surgery and is being used throughout the world to treat a wide spectrum of hepatic lesions. This advanced technique was introduced to overcome some of the limitations associated with laparoscopic liver surgery, but many of the techniques are not yet standardized [57]. The purported advantages of robot-assisted hepatic surgery include increased dexterity and enhanced suturing ability, which is in part due to a magnified three-dimensional view of the operative field, hand tremor filtration, and articulating instruments with seven degrees of freedom [57]. Furthermore, robot-assisted hepatic surgery is felt to significantly reduce surgeon fatigue, with improved performance during long operations. It is also felt that robot-assisted surgery facilitates the integration of new technologies such as a variety of intraoperative imaging modalities [57]. Acknowledged drawbacks include the absence of a dedicated instrument for liver transection, the need for additional surgeons and time for instrument replacement, the learning curve of the team to dock the instruments, and the lack of haptic feedback [57]. While it appears to be a promising technology, data to directly support its use are not abundant, especially comparing laparoscopic surgery and robot-assisted surgery. There is a definite paucity of data specifically evaluating robot-assisted hepatic surgery.

These authors reviewed 25 studies comparing laparoscopic and robot-assisted hepatic surgery [57]. They reported four sub-group analyses and found that data on the feasibility, safety, and oncologic effectiveness of robot-assisted hepatic surgery show that robot-assisted surgery reaches results comparable to laparoscopic surgery. Despite the increased cost and longer operating time, in certain situations, robot-assisted hepatic surgery allowed an increased rate of using a minimally invasive approach because of some of the specific benefits of a robot-assisted approach. They conclude that both open and minimally invasive surgery approaches should be provided, including the robot-assisted hepatic surgery, particularly for complex cases, which would otherwise be very demanding by laparoscopy. The technique used should be tailored to each patient, choosing the minimally invasive approach when possible, enhancing postoperative recovery.

There have been a number of reports about starting a new program for robot-assisted hepatic surgery. In a report of the first 50 robot-assisted hepatic operations, authors reported 50 procedures which represented 59% of all liver resections at a single institution [58]. This included 42/50 resections for malignancies and major liver resection in 16/50 (32%) of operations. They reported two patients who received a blood transfusion and one conversion to open liver resection. There was no mortality at 90 days postoperatively. The authors conclude that robot-assisted hepatic resection allowed minimally invasive resection of more difficult lesions.

Experience needed before starting a program in robot-assisted hepatic surgery has been discussed. In one institution, the authors report the concurrent implementation of laparoscopic and robot-assisted hepatic surgery programs. In a 5-year period, 92 patients underwent resection including 52 laparoscopic resections and 40 robot-assisted resections [59]. The incidence of complications (25% vs. 33%; *p* = 0.49), need for blood transfusions (2.5% vs.9.6%; *p* = 0.21), and median length of stay (6 vs. 5; *p* = 0.54) were similar in the laparoscopic and robot-assisted groups. The overall and recurrence-free survival at 1 and 5 years were similar in the two groups. The authors conclude that concurrent implementation of the two programs is feasible and safe.

As mentioned above, robot-assisted surgery offers the opportunity to integrate information from new imaging techniques [57]. Using a combination of pre-operative and intraoperative imaging is a novel approach, and some investigators have begun to incorporate artificial intelligence techniques in new concepts of robot-assisted hepatic surgery [60]. Imaging technologies such as Augmented Reality have been integrated to assist the surgeon and limit the intrinsic drawbacks of minimally invasive resections, such as the lack of tactile feedback, which can hamper numerous procedures during liver resection. The importance of haptic feedback is twofold in hepatic surgery. Firstly, from an oncologic standpoint, it helps locate the tumor and guide parenchymal transection. Secondly, haptic feedback aids the surgeon in being oriented in relation to intrahepatic landmarks. Arteries, veins, and biliary structures, present as thickened fibrotic sheaths, and the lack of haptic feedback when using a robot can fail to orient surgeons during dissection, which can result in vascular injuries. Augmented reality is effective in preoperatively planning strategy through 3D rendering and intraoperatively, targeting the lesion and resection margins [60]. Augmented reality-based intraoperative reconstructions and tracking systems may be used to map resection planes and show vascular structures during liver transection [60]. This is clearly just the beginning of efforts to integrate imaging technology into robot-assisted surgery.

Some of the issues with robot-assisted hepatic surgery have been mentioned above, including the difficulties associated with parenchymal transection, due to limitations of instrumentation. Perrakis and colleagues have developed a novel approach to parenchymal transection using three devices, including the monopolar scissors and bipolar Maryland forceps of the robot and a laparoscopic guided waterjet [61]. They reported a series of 28 patients who underwent this procedure for major (n = 12) and minor (n = 16) liver resections and referred to this method as a “3D parenchymal transection”. They reported operative time for major liver resections of 382 min vs. 252 min for minor resections (*p* < 0.01). Measured blood loss was 496 mL for major and 256 mL for minor liver resections (*p* = 0.090). The mean postoperative stay was 13.3 days for all patients. The authors achieved an R0 resection in all patients with malignancies. The authors conclude that this technique for parenchyma dissection in robot-assisted hepatic surgery is safe and feasible. This novel method allows for the controlled preparation of intrahepatic vessels and bile ducts. The combination of precise extrahepatic vessel handling with this novel technique of parenchyma dissection is a fundamental step forward for the standardization of robot-assisted hepatic surgery. This may be a significant contribution to teaching leading to wider adoption of robot-assisted hepatectomy [61].

There has been a slow evolution leading to the acceptance of minimally invasive hepatic resection. Over time, laparoscopic liver resection has become the standard of care for left lateral sectionectomy. The benefits of laparoscopic liver resection, including shorter operating and recovery times, less blood loss, and a lower incidence of postoperative adhesions, make this technique highly useful [62]. This approach has some limitations, such as restricted instrument motion, two-dimensional imaging, complex ergonomics, and unstable exposure. Robot-assisted hepatic surgery appears to offer solutions to some of these limitations of conventional laparoscopic surgery. As happened with initial laparoscopic liver surgery, the application of robot-assisted technology to liver surgery has not been smooth. The use of robot-assisted single port access in the early experience at one center has been reported [62]. These authors reported the first two cases of robot-assisted single-port access liver resection. These two procedures were accomplished with low blood loss and reasonable operating times. Their experience confirms that robot-assisted liver resection can be practical in select cases. Robot-assisted hepatic surgery with single-port access appears technically feasible and safe.

Robot-assisted hepatic surgery using a new platform, the da Vinci SP system (Intuitive Surgical, Sunnyvale, CA, USA), has recently been reported [63]. This system has three fully wristed and elbowed instruments and a flexible camera in a single 2.5 cm cannula. Using clamp crushing, locking clips, and an endoscopic linear stapler, these authors performed a left lateral sectionectomy using this innovative system through a single 3 cm incision. This report extends the application of single-port robot-assisted surgery to hepatic resections.

Another novel application of robot-assisted hepatic surgery is robot-assisted donor hepatectomy. This is especially important as living donor liver transplantation has become more widely performed. Minimally invasive donor hepatectomy has been reviewed, including robot-assisted living donor hepatectomy [64]. The first robot-assisted living donor hepatectomy was performed in 2012. This procedure has not become widely used for several reasons, but it is becoming more available at specialized centers. These authors reviewed several reports, including a review of 13 robot-assisted donor hepatectomies reported from Taiwan [64]. In comparison with open living donor hepatectomies, they found similar blood loss, complication rates, and donor recovery time. Another series reviewed compared 25 robot-assisted and 50 laparoscopic donor hepatectomies with comparable results. In another review, the authors evaluate results from centers worldwide comparing open, conventional laparoscopic, and robot-assisted donor hepatectomy [65]. These authors trace minimally invasive donor hepatectomy. They discuss the advantages of robot-assisted hepatectomy and emphasize that the primary advantage of robot-assisted surgery is that it mirrors open surgery rather than conventional laparoscopic surgery. The robotic platform also has the added benefits of overcoming a restricted range of motion, physiological tremor, and a limited field of vision. These benefits allow for meticulous tissue handling, precise dissection, and easier suturing. Finally, they emphasize the importance of a cautious approach in the adoption of these new methods.

The role of robot-assisted hepatic resection for the treatment of colorectal liver metastases was recently evaluated in a systematic review [66]. A total of 9 studies from 2008 to 2021 were selected, including a total of 262 patients (161 males, 97 females, 4 indeterminate), of whom 131 underwent simultaneous colon/rectal resections. The mean blood loss was 309 mL with a mean operating time of 251 min. The mean age was from 59 to 72 years, and BMI was 23.4 to 28.0 between the studies. The overall postoperative mortality was 0.4%, the mean length of hospital stay was 8.0 days, and the overall morbidity was 37%. The mean 3-year disease-free survival was 37%, and overall survival was 55%. This is the first large review of robot-assisted surgery for colorectal cancer liver metastases, and there are as yet no prospective randomized trials for this condition. The authors conclude that robot-assisted hepatic resection may be a technical upgrade for the minimally invasive approach to colorectal cancer liver metastases, including patients who undergo simultaneous operations.

Long-term oncologic outcomes after robot-assisted liver resections for primary hepatobiliary malignancies were reported in a multi-center study in 2018 [67]. This is an important study because the long-term oncologic outcomes have not been extensively reported to date. This study included 61 patients with hepatocellular carcinoma, cholangiocarcinoma, or gallbladder carcinoma who were analyzed retrospectively. Most of the resections were segmental resections (39%), and included central hepatectomies (18%), left lateral sectionectomy (15%), hepatectomy (13%), hemihepatectomy (13%), and right posterior segmentectomy (2%). The median hospital length of stay in this series was 5 days, and 7/61 patients required conversion of the procedure to open surgery. Complications occurred in seven patients with no perioperative mortality. Median 5-year overall survival was 56%, and disease-free survival was 38%. The authors conclude that robot-assisted hepatic tumor resection can be performed with long-term oncologic outcomes similar to data from open and laparoscopic resections.

There have been several cost-benefit analyses. The first is a propensity-matched retrospective study using data from the American College of Surgeons National Surgical Quality Improvement Program database, which compared 227 patients who underwent robot-assisted hepatic resection with a group of 227 patients who underwent laparoscopic liver resection [68]. Costs were assigned to perioperative variables, including operating room time, length of stay, blood transfusions, and complications. Costs were estimated using data from publicly available sources. In this matched cohort study, total costs of laparoscopic liver resection were USD 5.5M, and robot-assisted resections were USD 6.8M, an increase of 21%. The higher costs were mostly associated with length of stay and operating room time. Another study conducted a retrospective analysis of data from the 2015 National Readmission Database in the United States [69]. Three groups were evaluated using propensity-matched analysis of open, laparoscopic, and robot-assisted resections. Primary outcomes were six-month readmission rates and associated costs. This study included 3872 patients (open = 3420, laparoscopic = 343, and robot-assisted = 109). Robot-assisted liver resection had a lower 6 month readmission rate (18.3%) than laparoscopic (26.7%) and open (30%) resections. The robot-assisted approach was overall less expensive (USD 127,716.56 ± 12,567.31) than open (USD 157,880.82 ± 18,560.2) and laparoscopic resections (USD 152,060.78 ± 8890.13) with regard to total cost which includes readmissions. The authors found a financial benefit with robot-assisted resection in terms of the total cost, hospital length of stay, and readmissions in patients undergoing hepatic resection.

Indocyanine Green (ICG) is a simple and safe agent which has been used for some time for liver imaging. This agent allows for visualization of tumors, bile ducts, and liver parenchyma, and its use has been extensively reported in Japan and other Asian countries since 2008, but much less commonly in other parts of the world [70]. ICG can demonstrate anatomical liver segments by fluorescence angiography using direct or indirect tissue staining. Fluorescence cholangiography visualizes the intra- and extrahepatic bile ducts [70]. Specialized imaging techniques have been developed which facilitate intraoperative use of ICG. Given this, the use of ICG in minimally invasive hepatic surgery is of great importance. Franz and colleagues reported an initial experience with 18 patients who received ICG for intraoperative imaging of hepatocellular carcinoma, cholangiocarcinoma, peritoneal hepatocellular carcinoma metastases, adenomas, or colorectal liver metastases [70]. Of these, 7/18 were laparoscopic resections and 11/18 were robot-assisted resections. The authors reported a sensitivity for tumor imaging of 100%. In 27.8%, additional liver tumors were identified by ICG fluorescence. In 39%, a false positive signal could be detected, which occurred mainly in cirrhotic livers. Mehdorn and colleagues reported on 20 patients who underwent robot-assisted hepatic resections [71]. They point out that ICG may help compensate for the lack of haptic feedback in robot-assisted surgery and the benefits of an integrated near-infrared camera for imaging ICG. The authors conclude that ICG staining helped in most cases by detecting additional metastases or when performing an R0 resection. ICG has a limitation if it is given soon before surgery and in patients with severe cirrhosis. ICG staining may serve as a beneficial intraoperative aid in patients undergoing robot-assisted hepatic surgery. Given the increasing use of ICG in hepatic resections and the increasing number of minimally invasive resections, the use of ICG in minimally invasive hepatic resections is an important development in this field.

## 9. Comparative Studies

In this portion of the review, we will evaluate a number of studies that have been performed to compare the various approaches to hepatic surgery. While it is tempting to collect these studies in a table, careful analysis shows that these studies have considerable heterogeneity that does not lend itself to a collective analysis, and therefore each of these studies will be considered individually. There is no specific attempt here to be comprehensive, but rather studies that are representative have been selected.

A comparison of short- and long-term outcomes of 70 patients who underwent robot-assisted hepatic resections with a group of 252 patients who underwent open hepatic resections was reported [72]. Operative time was longer in the robot-assisted group (472 min vs. 349 min, *p* < 0.001), and estimated blood loss was lower in the robot-assisted group (269 mL vs. 548 mL, *p* = 0.009). Overall, the rate of postoperative complications among patients who underwent robot-assisted surgery was lower than that among patients who underwent open resection (31% vs. 58%, *p* < 0.001), although the rate of major complications was not different. Hospital length of stay was less for patients undergoing robot-assisted surgery (9.5 days vs. 15.1 days, *p* = 0.006). Among those patients with colorectal liver metastases, cholangiocarcinoma, and HCC, the overall and disease-free survivals were similar for the groups. The authors conclude that robot-assisted liver resections result in better overall outcomes with similar long-term oncologic outcomes when compared with open liver resections. Therefore, robot-assisted surgery is a viable option for minimally invasive major liver resections.

A meta-analysis of 39 studies including 2999 patients, was reported [73]. Of this group of patients, 1249, 1010, and 740 underwent robot-assisted, laparoscopic, and open hepatic resections. When they compared open and robot-assisted resections, operating time was greater in the robot-assisted group, but intraoperative blood loss, blood transfusion rate, rate of severe complications, and hospital length of stay were significantly reduced in patients who underwent robot-assisted hepatic resection. The overall incidence of complications was similar. In a comparison of laparoscopic and robot-assisted resection, they found longer operating time and greater blood loss in the robot-assisted group, but no differences in transfusion rate, incidence of complications, or hospital length of stay. The authors acknowledge the generally increased operating time with robot-assisted surgery and conclude that the robot-assisted approach is similar to laparoscopic resection and better than open resection.

A comparison of robot-assisted and laparoscopic surgery was reported to specifically look at results for left lateral sectionectomy [74]. The authors retrospectively compared perioperative data as well as postoperative outcomes of robot-assisted (n = 12) and laparoscopic (n = 31) left lateral segmentectomies. Indications for resection included malignant tumors (n = 31) and benign lesions (n = 12) including intrahepatic duct stones (n = 9). There were no significant differences in estimated blood loss, major complications, or lengths of stay, but operating time was longer in the robot-assisted group than in the laparoscopic left lateral sectionectomy (391 vs. 196 min, respectively) and the operating time for intrahepatic duct stones did not differ between groups (435 vs. 405 min, respectively; *p* = 0.190). Disease-free and overall survival of patients with malignancy did not differ significantly. The 2 and 5-year disease-free survival rates were 63 and 37%, respectively. However, robot-assisted left lateral sectionectomy costs were significantly higher than laparoscopic left lateral sectionectomy costs (USD 8183 vs. USD 5190, respectively; *p* = 0.009). The authors conclude that robot-assisted left lateral sectionectomy was comparable to laparoscopic left lateral sectionectomy in surgical outcomes and oncologic integrity during the learning curve. They further conclude that although robot-assisted left lateral sectionectomy was more expensive and time-intensive, it might be a good option for difficult indications.

A retrospective analysis of data from the Nationwide Readmissions Database was conducted to compare 45-day readmission rates for open, laparoscopic, and robot-assisted hepatic surgery [27]. The Nationwide Readmissions Database includes information from about 35 million weighted, including patients of all ages and payer types. The authors point out the advantage of using the National Readmissions Database, which does not limit readmission rates across state borders and therefore includes readmissions at institutions different from where the index operation was performed. A total of 11,186 patients were included in the analysis, of which 87.5% underwent open resections, 9.3% laparoscopic, and 3.1% robot-assisted. The authors conducted propensity score matching, after which the robot-assisted group included 331 patients and the open and laparoscopic groups had 662 patients each. The 45-day readmission rate was 13.5%, 12.9%, and 8.7% in the open, laparoscopic, and robot-assisted groups, respectively (*p* < 0.001).

A meta-analysis to evaluate the safety and efficacy of open, laparoscopic, and robot-assisted hepatic resections was reported [28]. The authors included 49 articles with 3702 patients, of whom 1901 (51%) had laparoscopic hepatic resections, 1741 (47%) had open hepatic resections, and 60 (1.62%) had robot-assisted resections. This study evaluated outcomes such as estimated blood loss, operating time, length of stay, status of resection margins, the rate of postoperative complications, perioperative mortality, and cost. There were no differences in operating times, surgical margins, or perioperative mortality among the three groups. Laparoscopic and robot-assisted resections showed no differences among all outcome measures. Compared with the patients who had minimally invasive resections, those undergoing an open resection had a higher estimated blood loss (mean net change 152.0 mL), longer hospital length of stay (mean difference, 2.22 days), and a higher total rate of complications (odds ratio, 0.5). Minimally invasive liver resections were found as safe as open resections and associated with lower estimated blood loss, shorter length of stay, lower complication rates, and similar rates of postoperative mortality. The authors conclude that there is no evidence-based advantage to robotic resection compared with laparoscopic resection.

A meta-analysis of 44 papers was conducted to examine the theoretical differences between open and laparoscopic hepatic resection as preparation for the Second International Consensus Conference on laparoscopic liver resection, which was held in 2014 in Morioka, Japan [11]. Hemihepatectomy was used as an example in this study, and 17 clinical questions stipulated in advance. Clinical questions that matched at least six articles were short-term results, long-term results, and cost, and important outcomes were isolated for each clinical question and compared between laparoscopic and open hepatic resections. Laparoscopic resection is favored for most outcomes. The authors caution that these comparisons are based on retrospective studies because there is no data from randomized controlled trials.

Given the accepted benefits of minimally invasive hepatic resection, the authors performed a comparison of laparoscopic and robot-assisted hepatic resections [75]. One of the important features of this review is that procedures were stratified by difficulty, and propensity score matching was performed. Databases from 6 high-volume centers were retrospectively reviewed, with a total of 936 laparoscopic and 403 robot-assisted hepatic resections included. High-difficulty procedures performed with robot-assisted techniques had lower blood loss, fewer blood transfusions, and lower conversion rates than laparoscopic resections, while laparoscopic resections were associated with lower postoperative morbidity and fewer complications. For intermediate and low difficulty resections, the intraoperative advantages of robot-assisted techniques gradually decreased to nonsignificant and laparoscopic techniques were associated with lower postoperative morbidity. The authors conclude that robot-assisted hepatic resections have neither surgical nor clinically significant benefits over laparoscopic hepatic resections for low-and intermediate-difficulty resections. Robot-assisted resections support difficult resections, possibly extending the indications for minimally invasive hepatic resections. It is hoped that future studies will be designed with stratification by the difficulty of the procedure to further investigate this finding.

A comparison of long-term oncologic outcomes for laparoscopic and robot-assisted resection of metastatic colon cancer lesions was reported using data abstracted from 6 high-volume centers in the USA and Europe, which includes 115 patients who underwent robot-assisted and 514 patients who underwent laparoscopic resection of colorectal cancer hepatic metastases [67]. Outcomes including mortality, morbidity, reoperation rate, readmission rate, intensive care unit admission requirement, hospital length of stay, and margin status were not statistically different between the two groups. Propensity score matching demonstrated similar overall survival and disease-free survival between robot-assisted and laparoscopic hepatic resections at 5 years (61 vs. 60% overall survival, *p* = 0.87, and 38 vs. 31% disease-free survival, *p* = 0.25 for pre-matching and similar results for post-matching). The authors conclude that propensity score matching of data from a large, multicenter database shows that robot-assisted hepatic resection of colorectal metastases is feasible and safe, with oncologic outcomes and survival similar to results with laparoscopic hepatic resections.

Investigators performed a meta-analysis of 485 patients from 8 studies to compare outcomes from robot-assisted and laparoscopic major hepatectomies, looking at preoperative settings, operative outcomes, and postoperative outcomes [76]. Compared to laparoscopic hepatic resections, robot-assisted major hepatectomies were associated with a significantly lower rate of conversion to open surgery and estimated blood loss. In this analysis, patients who underwent laparoscopic hepatic resections had shorter postoperative hospital lengths of stay compared to those who had robot-assisted hepatic resection. The authors conclude that the two surgical approaches provide similar results for major hepatectomy, with the exception of the outcomes stated above.

In another review, investigators compared robot-assisted and laparoscopic hepatic resections specifically for left lateral sectionectomy [77]. The authors determined the effects of surgical approach and complexity on postoperative length of stay and costs using univariate and multivariate analysis. A total of 258 patients underwent left lateral sectionectomy using robot-assisted or laparoscopic approaches. The laparoscopic approach was associated with similar outcomes and decreased costs compared to robot-assisted resection for ordinary complexity cases, while robot-assisted resection was associated with shorter postoperative length of stay for complex cases. Multivariate analysis showed robot-assisted left lateral sectionectomy was associated with a shorter postoperative length of stay but also predictive of higher costs. The authors conclude that there is no clinical benefit to robot-assisted left lateral sectionectomy compared to laparoscopic resection in cases of ordinary complexity, but that robot-assisted left lateral sectionectomy has potential advantages in selected complex cases. This result is similar to that reported by other investigators who looked at a greater variety of procedures and was described above [75].

## 10. Long-Term Outcomes

Oncologic outcomes after robot-assisted hepatic surgery are perhaps most important to patients. One study specifically reported long-term oncologic outcomes of robot-assisted hepatic surgery. An international multi-center retrospective study of 61 patients who underwent robot-assisted hepatic resection of a variety of malignancies was performed [67]. Age, gender, histology, margin status, extent of resection, disease-free survival, and overall survival were analyzed. Of the 61 included patients, 34 (56%) underwent robot-assisted resection for hepatocellular carcinoma, 16 (26%) for cholangiocarcinoma, and 11 (18%) for gallbladder cancer. Most resections were non-anatomical or segmental resections (39%), followed by central hepatectomy (18%), left lateral sectionectomy (15%), left hepatectomy (13%), hemihepatectomy (13%), and right posterior segmentectomy (1.6%). The median hospital length of stay was 5 days, and conversion to open surgery was performed in 7 patients (12%). There was no perioperative mortality. Median follow-up was 75 months, and 5-year overall survival and disease-free survival were 56% and 38%, respectively. The authors conclude that robot-assisted liver resection can be performed for primary hepatobiliary malignancies with long-term oncologic outcomes comparable to previously reported open and laparoscopic data.

## 11. Future Technology for Robot-Assisted Hepatic Surgery

There have been several reports on the future of robotic surgery, which include not only robot-assisted surgery but also surgery for which robots have increasing levels of autonomy. The current status of laparoscope-holder robots, master–slave robots, and hand-held robotic forceps has been reviewed in depth by Kawashima et al. [78]. These authors point out the wide variety of applications where robots are being used, such as robot assists for eye surgery, micro-surgery, catheter insertion, minimally invasive surgery, and scope holders. Hand-held robotic forceps are reviewed, including those that are mechanical as well as those that are actuator-driven. They identify current challenges in robotic surgery, including size and cost, haptic feedback, single port and natural orifice surgery, telesurgery, augmented reality, task automation, and coupling of a cyber–physical system with robots. This is a wide range of important topics that will be fertile ground for many years of research in the future.

Throughout this review, we have talked about robot-assisted surgery to differentiate it from robotic surgery, where the robot can perform tasks autonomously. One such specific task that has been investigated is suturing. Leonard and colleagues describe the Smart Tissue Anastomosis Robot (STAR), which is a vision-guided robotics system for laparoscopic suturing [79]. To date, STAR has been used in the laboratory only. STAR is a vision-guided system with a laparoscopic device that can place sutures using image-based commands. STAR is based on a commercial laparoscopic device attached to a motorized stage. The STAR system allows a surgeon to select and track incisions and the placement of individual sutures. The interface provides a *manual* mode that allows a surgeon to specify the exact placement of a suture and an *automatic* mode that automatically computes equal spacing of sutures based on incision shape.

The STAR system has been further developed with a new three-dimensional endoscope, a novel suturing tool, and a suture planning strategy for autonomous suturing [80]. The accuracy and consistency of the system were evaluated and compared to sutures placed manually by surgeons. The in vitro results show that STAR can reach 2.9 times better consistency in suture spacing compared to manual suturing and eliminate the need for suture adjustments. The consistency of suture pitch obtained by STAR matches those obtained by manual suturing.

Further development of STAR includes a novel three-dimensional path planning algorithm that facilitates the creation of a semi-autonomous robotic anastomosis on deformable tissue [81]. This algorithm incorporates continuous detection of three-dimensional near-infrared markers placed on the deformable tissue prior to the procedure, generating consistent suture placement using three-dimensional path planning based on the locations of the near-infrared markers and updating the suture plan after each completed stitch, which accounts for tissue deformation. The path planning algorithm was tested by comparing the anastomosis of a synthetic vaginal cuff by STAR and a surgeon. Results show that STAR achieved 2.6 times better consistency in suture spacing and 2.4 times better consistency in suture pitch than when performing a manual anastomosis. The STAR system is of great interest and will surely continue to develop and may eventually be used clinically. Certainly, the techniques developed by these investigators will be widely applicable to new systems as they are developed.

Another area of new technology applied to hepatic surgery is the concept of intraoperative navigation. The role of ICG in this rapidly expanding field has been discussed above. Preoperative simulation systems are widely available for planning hepatic surgery. Virtual reality, augmented reality, and mixed reality systems, all forms of extended reality, are being widely used for intraoperative navigation during hepatic surgery, especially with the recent rapid advancements in computing hardware and software [82]. These systems allow the surgeon to feel “immersed” in the operative field intraoperatively. One of the limitations of these systems relates to the ability to register the shape of the liver, especially with intraoperative deformations. Augmented reality allows 3D reconstruction, registration, tracking, and display and is being applied to hepatic procedures, including laparoscopic surgery using augmented reality assisted laparoscopic resection [83]. Augmented reality allows visualization of blood vessels and tumors in the liver during surgery, facilitating precise navigation during complex procedures. Liver shape transformation and registration errors intraoperatively were seen as the main factors that limit the application of this exciting new technology.

The combination of 3-dimensional imaging and robot-assisted surgery has been described in detail, particularly as related to hepatic surgery [84]. The authors point out the advantages of robot-assisted surgery as supported by advanced imaging techniques, which result in increased operative precision. The authors also discuss surgical navigation, which refers to the use of imaging equipment and image processing methods to visualize the patient’s imaging data before surgery. This allows the surgeon to precisely match the patient’s anatomy during surgery using alignment procedures and to display the position of surgical instruments in real-time. The accuracy of the tracking technique is important for the reliability of navigation. The accuracy of the tracking system depends in large part on the performance of the navigation system. Optical tracking systems (OTS) and electromagnetic tracking systems (EMT) are the two main tracking techniques in use today [84].

## 12. Conclusions

This review has described some of the applications of minimally invasive surgery (both laparoscopic and robot-assisted) in the treatment of hepatic lesions. This has been conducted as a historical review, beginning with early approaches to open hepatic surgery, then proceeding to laparoscopic liver surgery, and finally to robot-assisted surgery for the treatment of patients with liver disease.

There is great interest in comparing results among open liver surgery, laparoscopic resection, and robot-assisted surgery, although randomized prospective trials have not been reported. These trials are difficult to complete with adequate power to give meaningful results. There are few reports of comparative costs of the procedure. Data from multiple international centers are needed, using a system of accessible registries. Training programs are needed to teach these techniques. It is expected that some of these activities will be coordinated by the International Laparoscopic Liver Society [30]. Much of the data describing the advantages of robot-assisted surgery are from the point of view of the surgeon, not the patient. Minimally invasive surgical (both laparoscopic and robot-assisted) techniques for the treatment of liver lesions are evolving with international support.

Improving many aspects of robot-assisted surgery will depend on the combined efforts of surgeons and Biomedical Engineers. The engineers must acquire an understanding of what is important to surgeons, and surgeons must understand what is possible within the constraints of technology. An example of this is instrument motion and the number of degrees of freedom available for motion. Further improvements in instrument motion with more degrees of freedom may lead to more useful instrumentation [81]. The lack of sufficient haptic feedback is a longstanding issue in laparoscopic surgery and is even more lacking in robot-assisted surgery. Improved visualization is another goal of importance to surgeons that will require more input from biomedical engineers. Improved data integration will allow surgeons to view imaging studies intraoperatively, using augmented reality. Biomedical engineers are key players in integrating this new technology.

The meaning of the results presented in this review of liver surgery must be evaluated from the perspective of an individual patient who was recently told that they have a hepatic lesion. The patient surely seeks the best treatment to maximize their chances for meaningful long-term survival. In a review of robot-assisted prostate resection, the authors stated, “Patients should choose an experienced surgeon they trust and with whom they have a rapport, rather than basing their decision on a specific surgical approach” [42,54]. A recent review concluded with a similar statement for robot-assisted surgery of the pancreas [1]. A similar conclusion can be made for surgery of the liver, based on the data presented here. If the operation is performed open, laparoscopically, or by robot-assisted techniques, the long-term oncologic outcomes will likely be similar. Patients naturally expect a surgeon to use the approach that the surgeon judges to be most suitable. Procedures performed laparoscopically or as robot-assisted surgery may be associated with a slightly shorter hospital length of stay and lower intraoperative blood loss compared with open surgery, but the patient also expects that long-term outcomes will be similar. Studies reviewed here show that short-term outcomes, including complication rates, slightly favor laparoscopic and robot-assisted surgery, but there is still no conclusive data. The patient should seek to find the best (experienced) surgeon with whom they have a good rapport to conduct the operation at the best possible institution in an environment allowing them to have a beneficial therapeutic relationship and recover as quickly as possible. Patients who need treatment for lesions of the liver should not be concerned about the details of the surgical technique used. A comparative review suggests that there may be differences in outcomes based on procedure difficulty [75,77]. Laparoscopic and robot-assisted surgery may be similar for low- and intermediate-difficulty surgery, while the robot-assisted approach may have improved results for high-difficulty procedures. This idea will require further study to be verified.

The step-by-step, relatively controlled growth of robot-assisted hepatic surgery, in contrast to the situation which followed the introduction of laparoscopic cholecystectomy, has facilitated the identification of areas for improvement, some of which are at the nexus of medicine and biomedical engineering. Further developments in robotic and robot-assisted surgery which provide clear benefits to the patient will depend on the cooperative efforts of engineers and clinicians. It is difficult at present to demonstrate an objective benefit to patients from undergoing robot-assisted surgery [37], but some studies have shown benefits when difficult procedures are performed [75,77]. Stratification by difficulty may allow the identification of procedures, which will have an evidence-based benefit from the use of robot-assisted approaches.

## Data Availability

Not applicable.
